# Standardising Mental State Documentation on an Acute Psychiatric Ward Using the MINDY Framework

**DOI:** 10.1192/bjo.2026.11648

**Published:** 2026-06-30

**Authors:** Alba Garcia-Melendez, Chania Lambrinudi, Kishen Guruparan, Harith Ali, Kazuya Iwata

**Affiliations:** 1 East London NHS Foundation Trust, London, United Kingdom; 2 Queen Mary University of London, London, United Kingdom

## Abstract

**Aims:**

Consistent and comprehensive documentation is essential for patient safety, continuity and appropriateness of care, and effective communication on psychiatric inpatient wards. The Mental State Examination (MSE) remains the cornerstone of psychiatric assessment; however, routine nursing documentation often varies in structure, emphasis, and completeness. In other clinical settings, structured documentation tools have shown improved consistency and adherence to best-practice standards. Within psychiatric services, there is limited evidence examining whether routine nursing notes consistently capture the breadth of the MSE.

This case-note review focuses on documentation quality using MINDY, a structured mental state framework developed to support systematic inpatient assessment.

We aimed to compare the completeness and consistency of routine nursing inpatient psychiatric documentation with contemporaneous assessments completed using the MINDY framework.

We hypothesised that the former do not consistently show all core domains of the MSE and that MINDY provides a more comprehensive representation of the patient’s overall mental state.

**Methods:**

We designed this as a service evaluation and case-note review on an acute adult psychiatric inpatient unit. During a two-week period, selected randomly, three different resident doctors assessed all patients under a single consultant using the MINDY tool. We obtained corresponding nursing documentation for this period and compared these sources of information, only including days when both were available.

An independent medical student, not involved in patient care, performed the comparative analysis.

All records were anonymised prior to analysis. Local NHS information-governance procedures and General Data Protection Regulation (GDPR) were followed.

**Results:**

Routine nursing documentation demonstrated a median MSE-domain completeness of 40% across patients, with consistent focus on behaviour, activity, mood and cognition but near-complete omission of speech, thought process and insight.

In contrast, the MINDY framework structurally mandates assessment of nine of ten classical MSE domains, yielding a median completeness of 90% per assessment. The median difference in domain coverage was therefore 50 percentage-points. The magnitude of this effect was very large (Cohen’s h=1.13), and the difference was highly statistically significant on two-proportion testing (p < 0.001).

**Conclusion:**

Compared with routine narrative nursing documentation, MINDY more than doubled the breadth of mental-state recording, increasing median MSE-domain coverage from 40% to 90%. The size of this improvement was extremely large and statistically robust, supporting MINDY’s role as a powerful standardisation intervention rather than merely a symptom-rating scale.

These findings indicate that structured daily use of MINDY could enhance multidisciplinary communication and reduce systematic omission of key psychopathological domains on acute wards.

